# Treatment and outcomes of dogs with hepatocutaneous syndrome or hepatocutaneous‐associated hepatopathy

**DOI:** 10.1111/jvim.16323

**Published:** 2021-11-25

**Authors:** John P. Loftus, Adam J. Miller, Sharon A. Center, Jeanine Peters‐Kennedy, Michael Astor

**Affiliations:** ^1^ Department of Clinical Sciences Cornell University, College of Veterinary Medicine Ithaca New York USA; ^2^ Department of Biomedical Sciences Cornell University, College of Veterinary Medicine Ithaca New York USA; ^3^ Present address: Northstar Vets 315 Robbinsville‐Allentown Road, Robbinsville New Jersey USA

**Keywords:** hepatic disease, internal medicine‐canine, metabolic disease

## Abstract

**Background:**

Superficial necrolytic dermatitis (SND) in dogs is a rare disorder most commonly associated with hepatocutaneous syndrome. Although often reported as fatal, sporadically reported long‐term remissions might be more common than previously believed and linked to treatment regimens.

**Hypothesis/Objectives:**

Evaluate treatments and associated outcomes in dogs with hepatocutaneous‐associated hepatopathy (HCH) with or without SND, designated collectively aminoaciduric canine hypoaminoacidemic hepatopathy syndrome (ACHES).

**Animals:**

Forty‐one dogs of various breeds and ages diagnosed with ACHES.

**Methods:**

Retrospective study. Electronic surveys, medical records (2014‐2019), and communication with veterinarians provided data. Three treatment categories were each dichotomized: IV amino acid (IV‐AA) infusions (≥2 vs <2), supplements including S‐adenosylmethionine (SAMe), arginine with ornithine, glutathione, lysine, proline, omega‐3 fatty acids, or zinc (≥3 vs <3), and diet type (home‐cooked vs commercial). Optimal treatment was defined as receiving ≥2 IV‐AA treatments, ≥3 nutritional supplements, and a home‐cooked diet.

**Results:**

Most dogs (29/41, 71%) received IV‐AA infusions (23/29, ≥2 infusions). Twenty‐one dogs (51%) were fed commercial diets; 17/41 (41%) were fed home‐cooked diets. Most dogs received SAMe (32/41, 78%) and a median of 3 supplements. In 4 dogs, HCH remission occurred. Overall all‐cause median survival time (MST) was 359 days, and disease‐specific MST was 557 days (range, 1‐1783 days). Optimally treated dogs (n = 9) lived significantly longer (MST, >1783 days, *P* = .02) than variably treated dogs (MST, 214 days).

**Conclusions and Clinical Importance:**

Optimized ACHES management can resolve SND and HCH and confer long‐term survival.

**Abbreviations**
AA: amino acid
ACHES: aminoaciduric canine hypoaminoacidemic hepatopathy syndrome
DM: diabetes mellitus
HCS: hepatocutaneous syndrome
HCH: hepatocutaneous‐associated hepatopathy
SAMe: S‐adenosylmethionine
SND: superficial necrolytic dermatitis

## 1 INTRODUCTION

Canine hepatocutaneous syndrome (HCS) is an uncommon metabolic condition associated with a unique hepatopathy. It is the most common cause of superficial necrolytic dermatitis (SND) in dogs, a syndrome of crusting, erythematous, and histologically unique skin lesions. Necrolytic migratory erythema was first reported in humans as a condition associated with glucagonomas in 1942, whereas veterinarians first described HCS as a dermatopathy associated with diabetes mellitus (DM) in 1986.^1^, ^2^ Hepatocutaneous syndrome is a complex disorder involving SND lesions, a distinct hepatopathy (hepatocutaneous‐associated hepatopathy, HCH), hypoaminoacidemia, and aminoaciduria.^3^, ^4^, ^5^, ^6^, ^7^, ^8^, ^9^, ^10^ We recently reported dogs with HCH, hypoaminoacidemia, and aminoaciduria that did not have SND lesions at the time of diagnosis. Therefore, we define dogs with HCS or HCH without SND lesions as aminoaciduric canine hypoaminoacidemic hepatopathy syndrome (ACHES).

Most reported outcomes for dogs with HCS are poor (ie, average survival of 3‐6 months from time of diagnosis),^3^, ^9^ despite sporadic reports of survival times >2 years.^11^, ^12^, ^13^ Superior outcomes for dogs with HCS classically are thought to be achieved by administering IV amino acid (IV‐AA) solutions.^4^ In conjunction with IV‐AA infusions, high protein diets, traditionally commercial diets supplemented with whey protein, have been a mainstay of treatment for dogs with HCS.^4^ More recently, combined administration of IV lipid with IV‐AA infusions was reported to manage HCS in a single dog for 24 months.^11^ We have observed improved remission and survival in dogs with ACHES fed high‐protein home‐cooked diets. A recent case report describing long‐term survival of a dog with SND prescribed a home‐cooked diet formulated by a veterinary nutritionist corroborates these observations.^13^ To our knowledge, a study comparing the impact of diet types, home‐cooked vs commercial, with outcome in dogs with HCS has not been reported.

The evolving knowledge and clinical management of dogs with ACHES warrants reporting current treatments and outcomes for this condition. Terminology used here, unless specified otherwise, is as follows: HCS refers to previous descriptions of the syndrome where all cases had skin lesions, SND refers to the unique skin lesions, or syndromically to non‐HCS related cases (eg, glucagonoma) of SND in humans or dogs, and ACHES refers to the collective syndrome reported in this case series.

The uncommon nature of this condition, coupled with other factors that impact treatment standardization (eg, lack of a clear standard, owner finances), constrain the ability to conduct prospective controlled trials. With these limitations, inherent to virtually all retrospective studies under consideration, the objectives of our case series were to: (a) document treatments provided to dogs with ACHES, (b) identify treated cases with resolution of HCH based on serial ultrasound imaging, hepatic histopathology, or both, (c) determine all‐cause, and disease‐specific survival of dogs with ACHES, and (d) assess the relationship of author‐defined optimal treatment (≥2 IV‐AA treatments, ≥3 nutritional supplements, and home‐cooked diet) to the disease‐specific survival of dogs with ACHES. These objectives support the primary goals of our study to report treatments provided to dogs with ACHES and their broad impact on outcomes to identify avenues of investigation that warrant further scrutiny.

## 2 MATERIALS AND METHODS

### 2.1 Case selection criteria

One author (JPL) identified all cases for inclusion from enrollment in ongoing studies investigating ACHES. Diagnostic criteria similar to those previously described were applied.^5^ A histologic diagnosis of ACHES required SND or HCH lesions present in skin or liver biopsy samples, respectively, as previously described.^3^, ^10^ A board‐certified pathologist, including 1 of the authors (JPK), provided histologic diagnoses. Additionally, 1 of the authors (SAC), a board‐certified internist with expertise in hepatic pathology, recruited cases with HCH lesions and reviewed all liver histology. A clinical diagnosis of ACHES lacking a histologic diagnosis required the following features consistent with HCS/ACHES: (a) marcroscopic appearance and distribution of skin lesions, (b) compatible clinical pathology findings,^14^ (c) a classic (Swiss cheese) nodular hepatic ultrasound pattern, and (d) confirmatory results of plasma and urine AA profiles. A reference veterinary laboratory conducted all amino acid profiles (Amino Acid Laboratory, College of Veterinary Medicine, University of California‐Davis, Davis, California) as previously described.^5^ Urine amino acid profiles were normalized against urine creatinine concentration. Patients were excluded from this case series if skin lesions never developed in dogs lacking a histologic diagnosis or if the only skin or liver histopathology results obtained for a patient were inconsistent with SND or HCH, respectively. The Institutional Animal Care and Use Committee of Cornell University approved the protocol (2017‐0094) for studies from which this case population derives.

### 2.2 Data collection

We sent an electronic survey (Data S1) to veterinarians or owners of identified cases. Data not obtained in the survey was retrieved from medical records and communication with veterinarians directly responsible for case management. Data included: signalment; body weight (initial examination); presence or absence of DM; skin lesions (none, mild, fulminant); hepatic ultrasound abnormalities; histology reports of skin or liver biopsies; type of diet fed (commercial or home‐cooked diet [ie, owner prepared with combinations of meat or other protein sources, carbohydrate sources, oils, and vitamin‐mineral supplements]); involvement of a veterinary nutritionist in diet formulation; the number, frequency, and type of IV‐AA and lipid infusions; and, specific dietary supplements. Diet details were not collected for the study. However, any home‐cooked diets formulated by 1 of the authors (JPL) were balanced using commercial software (Balance IT, Full Autobalancer). Dietary protein inclusion met or exceeded the adequate intake for adult maintenance of dogs with required nutrients recommended by the National Research Council, typically with a target of approximately 50% of metabolizable energy from protein (unless contraindicated by intercurrent disease).^15^ Prioritized HCS supplements included: S‐adenosylmethionine (SAMe, any formulation), arginine with ornithine, glutathione, lysine, proline, omega‐3 fatty acids, and zinc. One author (JPL) developed dosing recommendations for supplementing glutathione, SAMe, arginine, ornithine, proline, and lysine based on previously reported plasma and urine AA abnormalities.^5^ When available, sequential hepatic ultrasound images were obtained by, or under the supervision of, a board‐certified radiologist for longitudinal comparisons at the authors' institution. The earliest date of detected skin or liver lesions, biopsy sample acquisition, documented hypoaminoacidemia, or aminoaciduria, established day 0 for survival analyses. Survival (days) after diagnosis and cause of death (disease‐specific and all‐cause) also were recorded. Cause of death was determined by the veterinarian managing the case or as documented in medical records and details assessed by 1 author (JPL). Clinical remission, unless otherwise specified, refers to the resolution of cutaneous lesions. Hepatic lesion remission refers to the apparent resolution of ultrasound or HCH histologic lesions in cases with previously documented lesions.

### 2.3 Treatment categories and groups

For survival comparisons, dogs were dichotomized into 2 groups for each treatment assessed: dogs that received ≥2 infusions vs <2 IV‐AA infusions, dogs fed a home‐cooked diet vs commercial dog food, and dogs receiving ≥3 (median number) prioritized supplements vs <3 supplements. Optimally treated dogs received ≥2 IV‐AA infusions, a home‐cooked diet, and ≥3 prioritized supplements. Dogs that received any other treatment combination were designated as variably treated. The survival impact of lipid emulsion treatment was evaluated independently.

### 2.4 Statistical analysis

Proportions and percentages were used to describe categorical data. We assessed data normality using the default software normality tests that included the Anderson‐Darling, Agostino & Pearson, Shapiro‐Wilk, and Kolmogorov‐Smirnov tests. We reported continuous variables with non‐Gaussian distributions as median and range and normally‐distributed data as mean and SD. Kaplan‐Meier survival analyses evaluated disease‐specific (ie, attributed to ACHES/SND) and all‐cause (ie, any other cause) mortality using the Gehan‐Breslow‐Wilcoxon (reported *P* values) and Log‐rank tests between groups as indicated. Disease‐specific mortality was calculated by censoring deaths not attributed to ACHES/SND, whereas all‐cause mortality considered all death events. Dogs alive at the time of writing were censored. We conducted the above analyses using Prism (GraphPad, San Diego, California) 9.0 or more recent software, which also generated corresponding graphs. We conducted multivariate disease‐specific survival analyses with the cox proportional hazards regression test in Statistix 10.0 (Analytical Software, Tallahassee, Florida) that included demographic and treatment data. Age and weight were continuous variables, whereas sex was categorical. Treatments analyzed categorically (0 = absent or not administered, 1 = present or administered) were DM, lipid infusions, commercial diet, glutathione, arginine, proline, lysine, SAMe, zinc, and omega‐3 fatty acids. Amino acid infusions were categorized by number of infusions, with 0 = 0, 1 = 1, 2 = 2‐9, 3 = >10. The total number of supplements administered also was included. A *P* value <.05 established significance for all analyses.

## 3 RESULTS

Forty‐one dogs were included in the case series (Data S2, Table S1) and as described in a companion manuscript.^14^ Ten cases were treated at the authors' institution, and the remaining cases were managed at various primary care and specialty referral practices.

Most dogs (71%, 29/41) received IV‐AA infusions. Amino acid solutions included 8.5%, 10% (Aminosyn, Hospira, Lake Forest, Illinois; Travasol, Baxter Healthcare, Deerfield, Illinois), and in 3 dogs 3% (ProcalAmine, B. Braun Medical, Melsungen, Germany) formulations. All dogs treated at the authors' institution received 25 mL/kg of 8.5% to 10% solutions (depending on product availability) delivered over 6‐8 hours. One author (JPL) recommended a dose of 50 mL/kg over 8 hours for sites utilizing 3% solutions, but 25 mL/kg was administered in some cases. Seventeen dogs received concurrent IV lipid infusions (Intralipid 20%, Fresenius Kabi, Lake Zurich, Illinois). All reported routes of administration were through peripheral catheters or long‐line jugular or saphenous catheters. We observed only rare cases of thrombophlebitis with peripheral catheter usage. The intra‐ and intercase frequency of infusions ranged widely, from weekly to >6 months, reflecting treatment individualization that included remission status and client factors. For example, 1 received weekly infusions for approximately 5 months until remission, and then the interval was tapered to every 4‐6 weeks. Another dog received 2 infusions approximately 1 week apart, after which the clients chose to pursue subsequent infusions only when paw pad lesions recrudesced (every 3‐9 months).

Nutritional supplements included SAMe (32/41, 78%), arginine with ornithine (16/41, 39%), glutathione (12/41, 30%), lysine (6/41, 15%), and proline (14/41, 34%). The total number of prioritized supplements administered to cases ranged from 0 to 7 (median, 3). The brand, source, and bioavailability of miscellaneous supplements provided were not consistently documented. Some dogs also received omega‐3 fatty acid (22/41, 54%) and zinc (13/41, 32%) supplements. Other miscellaneous supplements reported included protein powders, Pet‐Tabs (Zoetis, Parsippany, New Jersey), milk thistle extract, glutamine, phosphatidylcholine, ursodiol, and vitamin E. Administration of raw egg was not reported.

Just over half of dogs (21/41, 51%) were fed commercial diets, whereas 41% (17/41) were fed home‐cooked diets. Brands and formulas of commercial diets and nutritional details of home‐cooked diets were not compiled in the study. No diet information was available for 3 dogs. A veterinary nutritionist (Diplomate of the American College of Veterinary Nutrition, or residency trained) formulated 16/17 home‐cooked diets.

Intervals between treatment initiation to SND remission were highly variable among dogs. For example, 1 dog (Table S1, case #19) received 13 combined IV‐AA‐Intralipid infusions at 7 to 10‐day intervals before achieving SND remission, whereas other dogs achieved substantial improvement with as few as 1 to 3 combined IV‐AA‐lipid infusions. Combined IV‐AA‐lipid infusions did not appear to accelerate cutaneous lesion resolution. The resolution of cutaneous lesions ostensibly preceded evidence of hepatic resolution.

**FIGURE 1:**
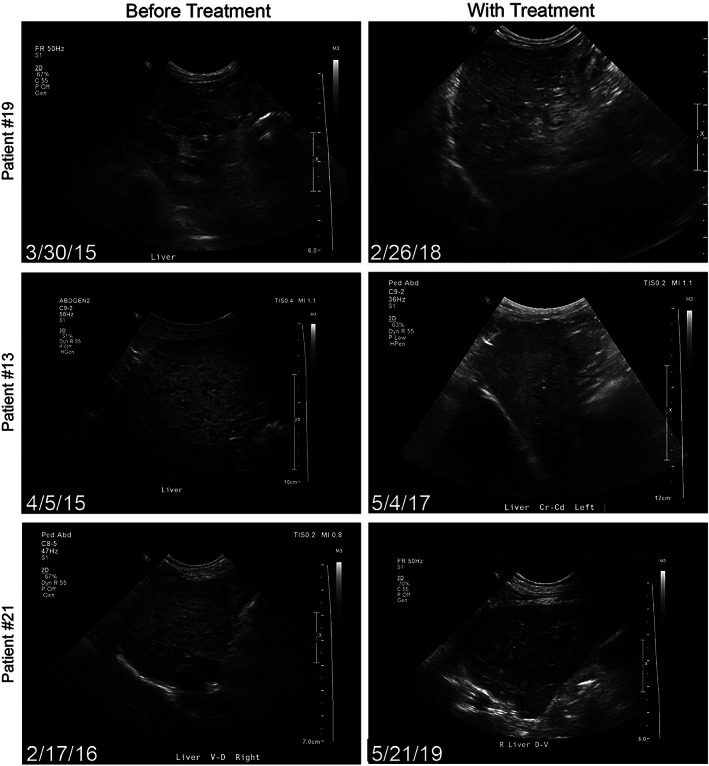
Liver ultrasound images from 3 dogs before and during aminoaciduric canine hypoaminoacidemic hepatopathy syndrome (ACHES) treatment. Each row of panels represents an individual dog. Panels on the left are ultrasound images of livers obtained at diagnosis for 3 dogs (corresponding patient numbers in Table S1). The right panels are ultrasound images of the same dogs during treatment. Dates are the day of ultrasound examinations

**FIGURE 2:**
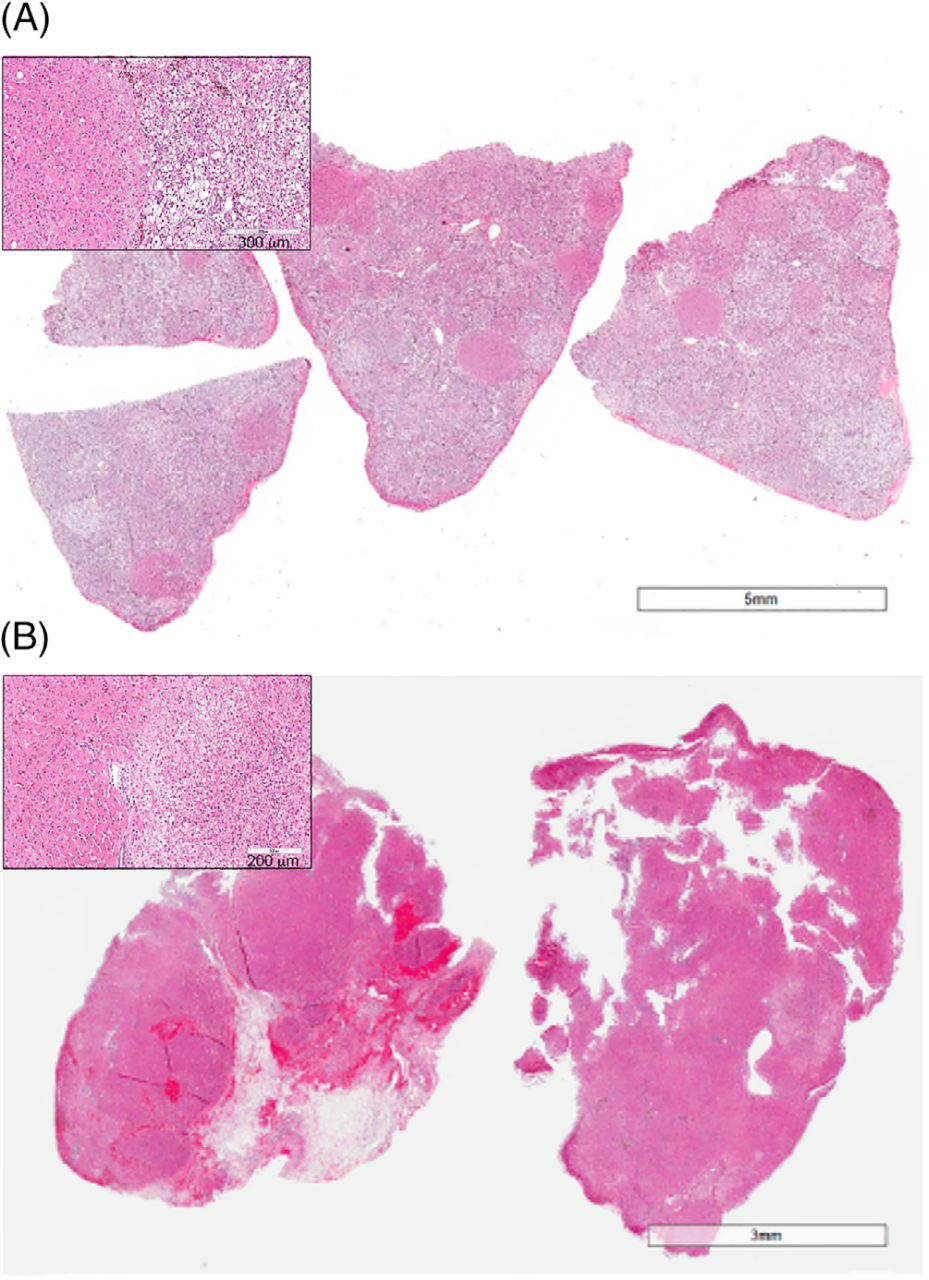
Histologic resolution of hepatocutaneous hepatopathy (HCH) in a dog (#21) with ACHES. (A) Photomicrograph of liver section (H&E staining) at the time of definitive diagnoses, higher magnification inset illustrates the classic moth‐eaten interface between a region of degenerative vacuolar hepatopathy and proliferative nodule. (B) Photomicrograph of liver section (H&E staining) from the same dog after 4 years of ACHE management, documenting resolution of HCH. The higher magnification inset illustrates the interface between a region of mild to moderate vacuolar change and adjacent parenchyma

Sequential ultrasound and histologic evaluations indicated HCH remission in 4 dogs (Table S1, cases #13, 19, 21, and 39) with long‐term survival ranging from 846 to 1783 days. Three dogs (Table S1, cases #13, 19, 21) had serial ultrasonographic images documenting hepatic architectural improvement, consistent with improvement in mitigation (Figure 1). These dogs received IV‐AA and lipid infusions weekly to quarterly (every 3 months) and were fed nutritionally balanced home‐cooked diets. Three dogs had diagnostic and posttreatment liver biopsies. Liver biopsy evaluation confirmed HCH resolution 4 years after initiation of continuous enteral arginine and ornithine, proline, and SAMe supplementation in 1 dog (Table S1, case #39). Liver lobectomy for hepatocellular carcinoma in another dog (Table S1, case #19) 4.5 years after HCS diagnosis provided an opportunity for sampling macroscopically unaffected tissue. These biopsy samples confirmed HCH remission, initially inferred from ultrasound imaging at the time of ACHES diagnosis. This dog initially was referred for severe SND and was managed using weekly to monthly IV‐AA and lipid infusions, enteral supplements (ie, glutathione, arginine with ornithine, proline, lysine, SAMe, zinc, and omega‐3 fatty acids), and a balanced home‐cooked diet to control cutaneous lesions. In a third patient (Table S1, case #21), cholelithiasis and suspected cholecystitis prompted exploratory laparotomy for cholecystectomy and liver biopsy 3.5 years after biopsy‐diagnosed HCH. This second biopsy sample confirmed HCH resolution but identified dysplastic foci (Figure 2). This patient had been managed with monthly IV‐AA and lipid infusions, enteral supplements (SAMe, arginine with ornithine, glutathione, proline, omega‐3 fatty acids, and zinc) and a balanced home‐cooked diet.

**FIGURE 3:**
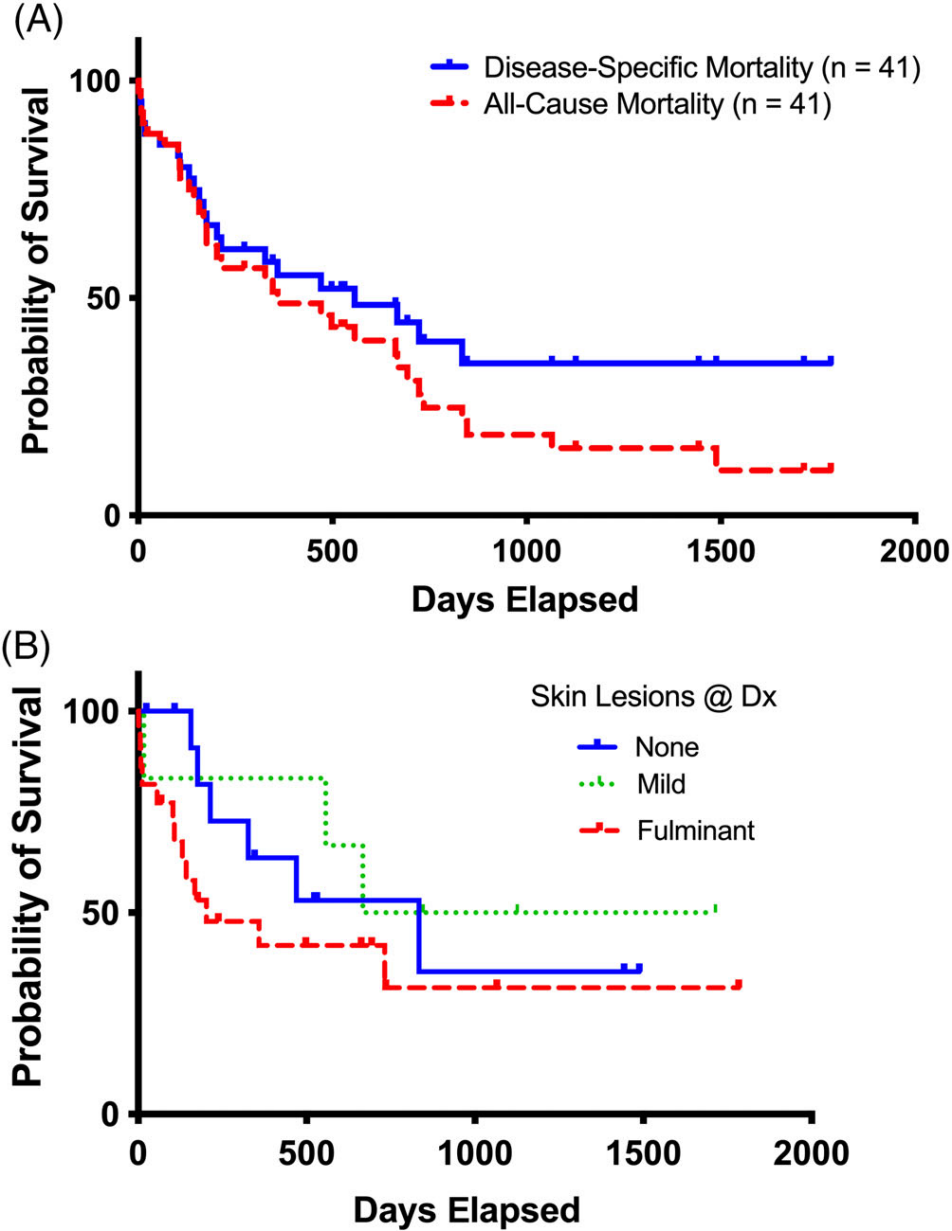
Survival of 41 dogs with aminoaciduric canine hypoaminoacidemic hepatopathy syndrome (ACHES) compared using the Kaplan‐Meier method. (A) Survival curves compare disease‐specific to all‐cause mortality. There was no significant difference between all‐cause or disease‐specific mortality (disease‐specific median survival, 557 days; all‐cause median survival, 359 days; *P* = .46). (B) Survival curves comparing ACHES cases by skin lesion status at diagnosis (none: median survival, 834 days; mild: median survival, 1191 days; fulminant: median survival, 203 days) disclosed no significant difference (*P* = .16). Legends indicate groups corresponding to survival curve lines. Tick marks indicate censored events

Survival data were available for all 41 dogs in the study, with 11 dogs alive or censored at the time of manuscript preparation. Of the remaining 30 dogs, 29 dogs were euthanized, and 1 dog died. Death in 67% (20/30) of dogs was attributed to ACHES, usually related to cutaneous lesions. Death in 33% (10/30) of dogs was attributed to other causes, prompting comparison of all‐cause mortality to disease‐specific mortality. Survival times ranged from 1 to 1783 days. Disease‐specific MST was 557 days, with 35% survival at 1783 days. Although the all‐cause MST (359 days; 10.3% survival at 1783 days) was numerically shorter than disease‐specific survival; the difference was not significant (Figure 3A). Because our cohort of dogs with ACHES diverges from reported cases of HCS by the presence or absence of skin lesions, we compared the survival of dogs by skin SND lesion status (none, mild, fulminant) at diagnosis. We found no statistical difference (*P* = .16) in survival between dogs based on skin lesion status (Figure 3B). Other clinical variables were included in a multivariate survival analysis. No significant relationship was found between survival and age or sex. Although weight was a significant survival variable, the relative risk (1.11) was small. Diabetes mellitus was associated with decreased risk (Table 1).

**TABLE 1:** Cox proportional hazards regression

Variable	*P* value	Relative risk	Lower CI	Upper CI
Age	.71	0.95	0.73	1.24
Sex	.73	1.27	0.33	4.92
Weight	.05*	1.11	1	1.22
Diabetes mellitus	.0003*	0.01	0	0.12
Amino acid infusion	.01*	0.39	0.18	0.82
Lipid infusion	.64	0.72	0.16	3.26
Commercial diet	.0007*	25.48	3.91	165.92
Gluathione	1	112.74	0	Infinite
Arginine	1	0	0	Infinite
Proline	1	4.52	0	Infinite
Lysine	1	251.67	0	Infinite
SAMe	1	0.05	0	Infinite
Zinc	1	3.96	0	Infinite
Omega‐3 fatty acids	1	0.55	0	Infinite
Number of supplements	1	0.67	0	Infinite

*Note*: *Indicates significant *P* values.

**FIGURE 4:**
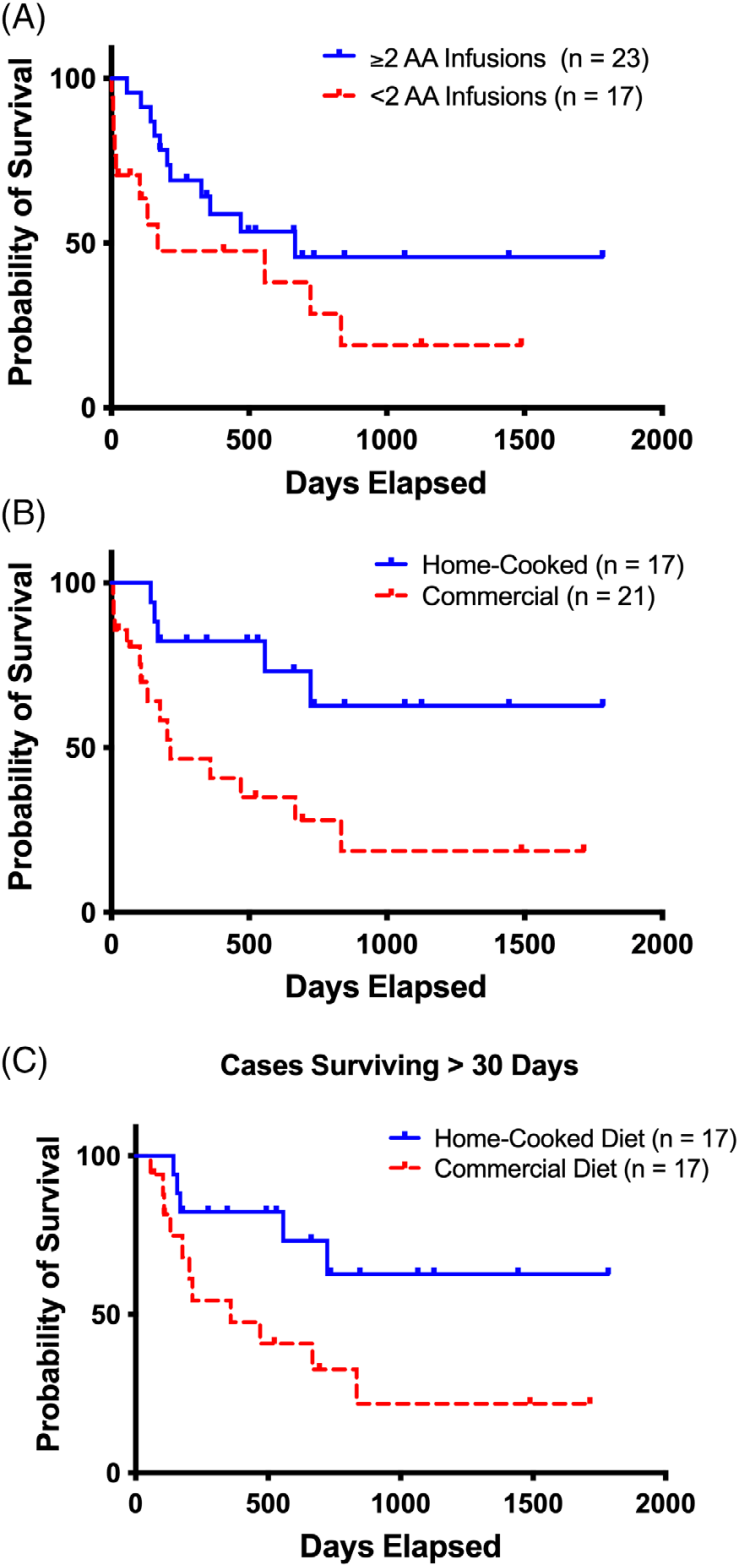
The relationship between infusion or diet treatments with disease‐specific survival of dogs with aminoaciduric canine hypoaminoacidemic hepatopathy syndrome (ACHES) using the Kaplan‐Meier method. (A) Survival curves comparing dogs receiving ≥2 (median survival, 667) days vs <2 (median survival, 168 days) amino acid infusions are significantly different (*P* = .05). (B) Survival curves of dogs fed home‐cooked (median survival, >1783 days) vs commercial diets (median survival, 214 days) are significantly different (*P* = .007). (C) Survival curves, excluding dogs surviving <30 days, comparing dogs fed home‐cooked (median survival, >1783) vs commercial (median survival, 359 days) diets are significantly different (*P* = .03). Legends indicate groups corresponding to survival curve lines. Tick marks indicate censored events

We assessed the relationship between survival and level of treatment independently for each treatment category. Dogs that received ≥2 IV‐AA infusions survived significantly (*P* < .05) longer (MST, 667 days; range, 56‐1783) than dogs receiving <2 IV‐AA infusions (MST, 168 days; range, 1‐1488; Figure 4A). No statistical difference was found in the survival of dogs that received lipid infusions vs not or <3 vs ≥3 prioritized enteral supplements. Feeding a home‐cooked diet was associated with significantly (*P* = .007) longer survival (MST, >1783 days; range, 143‐1783) than dogs fed commercial diets (MST, 214 days; range, 6‐1714; Figure 4B). Four dogs that ate primarily commercial diets died within 30 days of diagnosis. In contrast, the earliest death in a dog fed a home‐cooked diet occurred at 143 days. Even when the 4 dogs with short‐term survival were excluded from survival analyses, home‐cooked diets (MST, >1783 days; range, 143‐1783) were associated with significantly (*P* = .03) longer survival than commercial diets (MST, 359 days; range, 56‐1714; Figure 4C). Multivariate analysis corroborated the univariate survival findings (Table 1).

**FIGURE 5:**
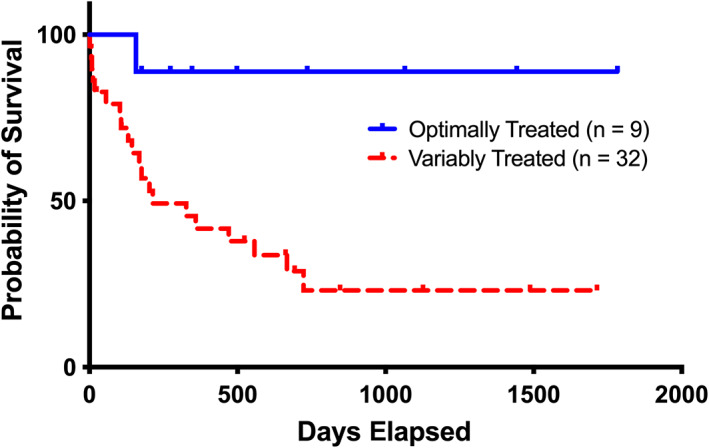
The relationship between treatment comprehensiveness and dogs' disease‐specific survival with aminoaciduric canine hypoaminoacidemic hepatopathy syndrome (ACHES). Survival curves comparing optimally‐treated dogs (receiving ≥2 IV‐AA treatments, ≥3 prioritized supplements, and home‐cooked diets) to all other, variably treated, dogs. Optimally‐treated dogs lived (median survival, >1783 days) significantly (*P* = .02) longer than variably treated dogs (median survival, 214 days). Legends indicate groups corresponding to survival curve lines. Tick marks indicate censored events

Disease‐specific survival analyses assessed the impact of author‐defined optimally combined treatments (Figure 5). Optimally treated dogs (received ≥2 IV‐AA infusions, ≥3 prioritized enteral supplements, and home‐cooked diets) survived significantly (*P* = .02) longer (MST, >1783 days) than variably treated (all other treatment combinations) dogs (MST, 214 days). Of the optimally treated dogs, only 1 death (at 157 days) was attributed to ACHES/SND.

## 4 DISCUSSION

**FIGURE 6:**
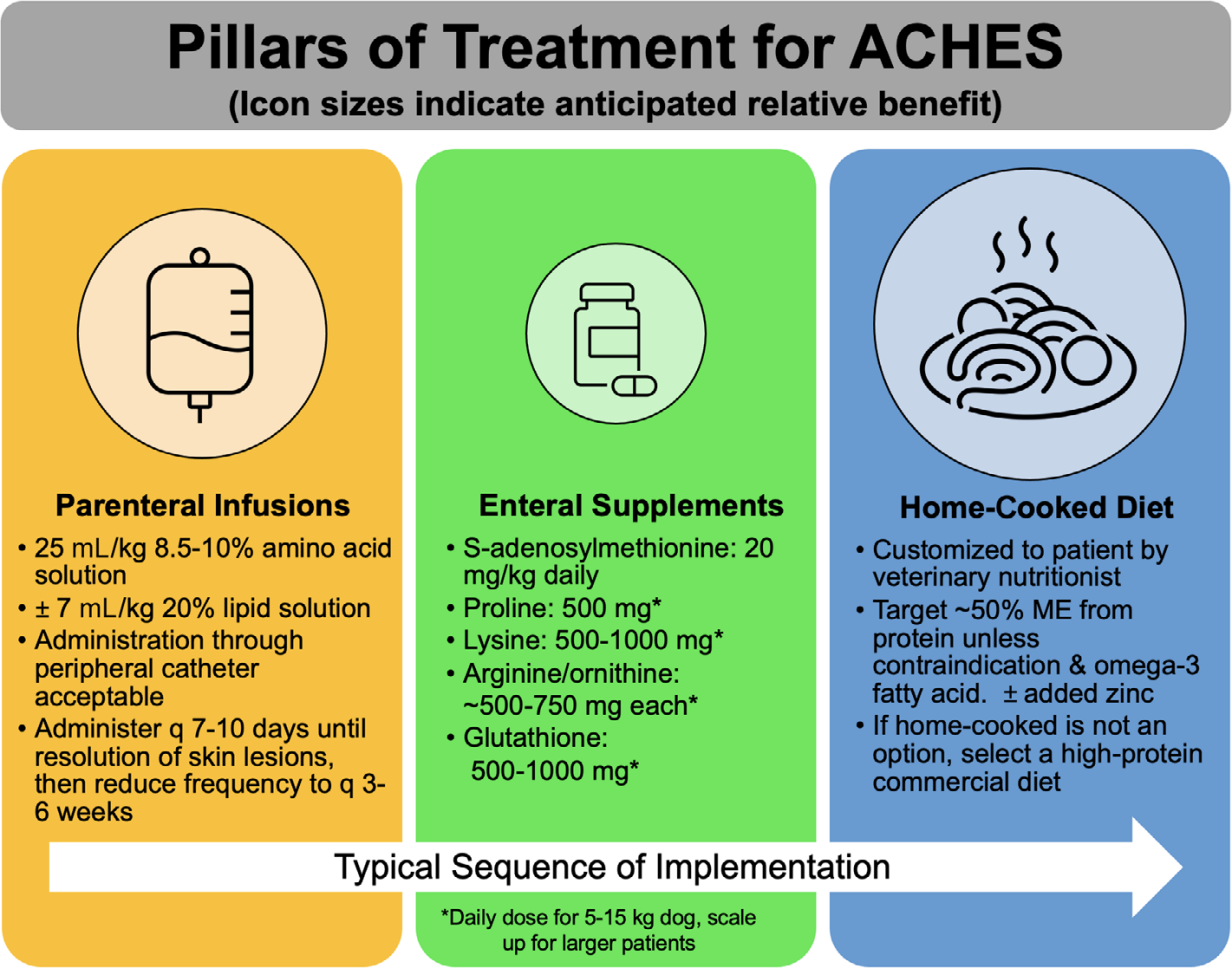
Author‐recommended treatment strategy for dogs with aminoaciduric canine hypoaminoacidemic hepatopathy syndrome ACHES. The typical sequence of treatment pillar implementation is not necessarily a recommended sequence and may be customized to each patient. ME = metabolizable energy. The PO supplemental zinc dose for HCS patients is 10 mg elemental zinc/10 lbs. body weight. Author supplement recommendations: S‐adenosylmethionine (SAMe, Denosyl or Denamarin, Nutramax Laboratories, Edgewood, Maryland), arginine/ornithine (PRO PERFORMANCE L‐ARGININE L‐ORNITHINE 2500, GNC, Pittsburg, Pennsylvania), glutathione (Glutathione 500 mg veg capsules preferred, NOW Foods, Bloomingdale, Illinois), lysine (L‐lysine, 500 mg veg capsules or Double Strength 1000 mg tablets, NOW Foods, Bloomingdale, Illinois), proline (L‐Proline 500 mg veg capsules, NOW Foods, Bloomingdale, Illinois)

We aimed to evaluate the outcome of dogs with ACHES and the impact of treatment on patient survival. We documented long‐term survival times for ACHES, with dogs achieving cutaneous and sometimes hepatic remission in response to a combination of IV‐AA (+/− lipid) infusions, targeted enteral supplements, and balanced home‐cooked diets. Indeed, 5 dogs survived >3 years. Therefore, implementing a multimodal approach can effectively achieve disease remissions that substantially prolong the lives of dogs with ACHES. These data and our experience support the recommendation of a “3‐pillar” approach to treatment (Figure 6).

Clinical resolution for patients with HCS historically has been assessed by inspection of SND lesion status. Improvement may be evident as early as 1 week after a single IV‐AA infusion or may be protracted. For example, in our study, 1 dog required 13 treatments, thus refuting the clinical impression that a predictable number of infusions indicates that an animal will not respond. Frequent monitoring of footpad and skin lesions is essential to guide treatment recommendations. Histology of liver biopsy specimens can directly assess the remission of HCH. Alternatively, resolution of nodular hepatopathy on ultrasonographic imaging implies HCH improvement. In addition to HCH resolution, we observed dysplastic hepatocellular foci in 1 dog and hepatocellular carcinoma in another. One of the authors (SAC) also has observed apparent neoplastic transformation in other dogs with HCS. Proliferative foci are pathognomonic for HCH and distinct from regenerative nodules.^3^, ^10^ It is possible that the distinctive proliferative foci in this syndrome could transition to dysplastic foci with evolution to neoplasia. Thus, HCH may predispose dogs to hepatocellular carcinoma. These observations prompt us to recommend periodic hepatic ultrasound imaging in dogs surviving >6 months.

Parenteral IV‐AA infusions have long been considered the gold standard treatment for dogs with SND.^3^ We did not record individual IV‐AA infusion protocols because we routinely recommend 25 mL/kg (8.5%‐10% AA solutions) over 6‐8 hours.^11^ Additionally, we typically recommend at least 2 infusions a week apart at treatment initiation. This recommendation, and the desire to avoid survival bias in patients that received high numbers of infusions, informed our infusion dichotomization cut‐off. In other words, dogs could have received more infusions because they lived longer. This caveat still applies and must be considered when interpreting relationships that may not indicate causality. Although AA infusions conferred a survival benefit on dogs in our study beyond published expectations,^9^ the response was less robust than anticipated and relatively unpredictable. Fatty acid deficiencies occur in some humans with SND and respond to combined AA and lipid emulsion infusions.^16^ Such lipid emulsions (typically 20% solutions) provide essential fatty acids, including linoleic acid and alpha‐linolenic acid, and phospholipids found in cell membranes.^17^ To our knowledge, fatty acid deficiencies have not been documented in dogs with HCS. However, a single case report describes lipid infusion for a dog with HCS.^11^ Based on these reports, we typically include lipid infusions in the treatment protocol unless the patient is hyperlipidemic or has some other contraindication to their use. Although we did not observe an independent survival benefit from lipid infusions, other benefits could have been derived from this intervention (eg, undetermined metabolic effects). Therefore, we recommend individualizing infusions to each patient based on concurrent disease, patient contraindications, and clinical response.

Enteral supplements provided in our study strategically address lysinuria, prolinuria, and abnormally low plasma AA concentrations characteristic of ACHES.^5^ Of particular concern were supplements targeting depleted AAs essential for collagen synthesis (lysine, proline), supporting the urea cycle (arginine and ornithine), and for glutathione synthesis (cysteine, glycine, and SAMe).^18^, ^19^, ^20^, ^21^ S‐adenosylmethionine, a key intermediate in the 1‐carbon metabolism cycle, is an essential precursor to glutathione and is a vital methyl donor for essential transmethylation reactions. Methionine adenosyltransferase 1A (MAT1A) primarily mediates the hepatic synthesis of SAMe. Reduced MAT1A gene expression and enzyme isoform switching restrict SAMe synthesis in some liver diseases of humans and in rodent models.^22^ Although MAT1A has not been studied in ACHES, we recommend dietary supplementation with bioavailable SAMe to address this possible phenomenon. This approach is particularly convincing in animals with low plasma cysteine concentrations, as typically observed in SND and ACHES.^23^

Feeding a home‐cooked diet to dogs with ACHES conferred the greatest independent impact on patient survival. Our clinical experience predicated survival comparisons between dogs fed commercial vs home‐cooked diets. Similarly, long‐term (718‐day) survival recently was documented in a dog with HCS treated with IV‐AA infusions and a balanced home‐cooked diet.^13^ Like many dogs reported in our study, that dog eventually succumbed to an illness unrelated to HCS. The authors hypothesized that the abundance of lysine in the home formulated diet might have compensated for marked ACHES‐associated lysinuria. One of us typically targets a protein inclusion of approximately 50% of metabolizable energy (ME) for dogs with ACHES. This target is based on clinical experience because no data‐driven guidelines for dietary protein targets for this syndrome currently are available, and lower targets may be appropriate. Comparatively, a 30/20 (% protein/% fat, as fed) commercial performance diet, sometimes used for ACHES, typically provides approximately 30% of ME from protein. Provision of a higher protein allowance by using home‐cooked diets in ACHES might explain the survival benefit. However, the recently reported HCS dog managed with IV‐AAs and a home‐cooked diet (approximately 37% ME from protein), only modestly increased estimated dietary protein intake from the dog's previous commercial food (approximately 30% ME from protein).^13^ A possible explanation is that protein digestibility in commercial diets typically ranges from 80% to 90%, whereas protein digestibility in home‐cooked diets often exceeds 90%.^24^, ^25^ Alternatively, commercial diets rarely can be a source of hepatotoxic agents, such as mycotoxins. However, the histologic lesions of HCH are distinct from aflatoxin‐induced lesions, and no dogs with apparent hepatotoxin‐induced histologic lesions were found in this case series.^26^ Detailed nutritional analyses of home‐cooked diets fed to dogs with ACHES might better elucidate the basis for this survival benefit. Comparing various macronutrients in home‐cooked and commercial diets was beyond the scope of our study but is an important future objective.

We investigated all‐cause as well as disease‐specific survival. Reporting each metric helps clarify the impact of unrelated illnesses on survival. In our study, disease‐specific survival of dogs exceeded all‐cause survival for dogs with SND or HCS previously reported as >200 days.^3^, ^6^, ^7^, ^8^, ^10^, ^11^, ^12^ A crucial point in comparing our cohort of dogs to this historical population is that we chose to include dogs that did not have classic SND skin lesions. Thus, early recognition of cases likely contributed to the longer survival we observed in our case series. Certain drugs, specifically phenobarbital, are associated with HCS.^27^ Drug discontinuation in such cases could eliminate an inciting factor for ACHES, but in previous reports of SND or HCS discontinuation of phenobarbital did not influence survival times in affected dogs.^27^ Furthermore, although phenobarbital administration was not specifically evaluated in our case series, we did not observe any cases in which phenobarbital or other drug administration or discontinuation influenced patient outcomes. Interestingly, ACHES cases with DM had a decreased risk of mortality. It is unclear if this finding is related to the relatively small number of dogs with DM, or if there is a metabolic basis for this observed effect.

The inclusion of only 41 dogs in our study limited statistical power for some comparisons. The low incidence of ACHES in dogs impedes larger‐scale prospective studies needed to control confounding variables. We acknowledge that some biases may have influenced case outcomes, including financial concerns limiting optimized medical management in some dogs (resulting in short‐term survival). Another critical variable influencing case management was dependence on owner perception of illness severity. Although 1 author (JPL) devised all treatment recommendations, owner observations and decisions and other factors may have influenced treatment heterogeneity. Consequently, the independent benefit of any single treatment is difficult to assess. However, anecdotally we have observed dogs respond to AA infusions alone or home‐cooked diets alone, and single treatment approaches should be considered for cases in which combination treatment cannot be pursued for various reasons. Although more rigorously standardized treatment protocols are desirable, variabilities encountered in our study reflect the realities of clinical practice. Dichotomizing diets as commercial or home‐cooked is a simplification of complex nutritional considerations that warrant further investigation. Future studies are needed to determine if home‐cooked diets are superior to commercial diets in managing this condition. Detailed nutritional analyses in the future could inform ideal commercial diets should the optimal dietary nutrient composition for ACHES cases be elucidated. Owner financial concerns and some owners' ability to travel to a specialty hospital influenced some treatment decisions. Causes of death not attributed to ACHES/SND included unequivocal conditions, such as pneumonia and septic peritonitis (Table S1), supporting the conclusion that a substantial proportion (approximately 33%) of our case fatalities was unrelated to ACHES/SND. Some non‐ACHES/SND causes of death could have been attributable to an as yet unidentified syndromic pathophysiology.

We propose a combination treatment standard‐of‐care for dogs with ACHES (Figure 6) that substantially prolongs the lifespan of affected dogs based on our collective clinical experience and results of our study. That HCH and SND lesions can resolve with treatment suggests a shared metabolic or cellular trigger that causes ACHES's clinical features. Elucidating ACHES's etiopathogenesis will improve early recognition and treatment strategies for this syndrome beyond that described here. The ability to achieve remission and long‐term survival in dogs with ACHES, even those with classic SND lesions, should be considered by clinicians when counseling clients regarding ACHES treatment options and prognosis.

## CONFLICT OF INTEREST DECLARATION

Authors declare no conflict of interest.

## OFF‐LABEL ANTIMICROBIAL DECLARATION

Authors declare no off‐label use of antimicrobials.

## INSTITUTIONAL ANIMAL CARE AND USE COMMITTEE (IACUC) OR OTHER APPROVAL DECLARATION

Cornell University IACUC approval, protocol 2017‐0094, was affiliated with the studies recruiting cases but was not needed specifically for this data collection.

## HUMAN ETHICS APPROVAL DECLARATION

Authors declare human ethics approval was not needed for this study.

## Supporting information


**Data S1**: Supplementary File 1.

**Data S2**: Supplementary File 2.

**Table S1**: Patient demographics, clinical features, and survival information.
