# Supplementary material for: Treatment and outcomes of dogs with hepatocutaneous syndrome or hepatocutaneous‐associated hepatopathy
Source: J Vet Intern Med. 2021 Nov 25;36(1):106–15. doi: 10.1111/jvim.16323 (PMC8783367; doi:10.1111/jvim.16323)
Supplement: Supplementary file 1 — Data S1: Supplementary File 1. [file JVIM-36-106-s003.pdf]

Supplementary File 1

Online Questionnaire

1. Email address
2. Client first and last name
3. Pet's name
4. Patient breed
5. Sex of patient
    - female spayed
    - male neutered
    - intact female
    - intact male
6. Date of birth
7. Date diagnosed with hepatocutaneous syndrome.
8. Patient weight (in kg)
9. How was the patient's diagnosis obtained? (Please note: you may choose skin and liver biopsy if both were performed, but can otherwise only check one answer.)
10. How would you describe skin lesions at the time of diagnosis?
    - No [None]
    - Questionable [Mild]
    - Yes [Fulminant]
11. Was the pet diabetic at the time of diagnosis?
    - Yes
    - No
12. If not, did the patient subsequently develop diabetes mellitus?
    - Yes
    - No
13. Has the patient ever had a liver mass?
    - Yes
    - No
14. If the patient had/has a liver mass or masses, please provide the histologic diagnosis if it was obtained.
    - Hepatocellular carcinoma
    - Other (indicate)
15. Is the patient currently alive?
    - Yes
    - No
    - Other [e.g., unknown, lost to follow-up]
16. Date euthanized or diet, or current date if still alive.
17. If the patient was euthanized was this because of HCS or due to another reason? If for another reason, please explain.

18. What therapy did the above patient receive (can select multiple options)?
    - IV Amino Acid
    - IV Lipid
    - Home Cooked Diet (formulated by veterinary nutritionist)
    - Home Cooked Diet (not formulated by veterinary nutritionist)
    - Commercial High Protein
    - Other
19. If receiving IV amino acids or IV lipids please list specific formulation.
20. How many parenteral treatments (amino acid +/- lipid infusions) has this patient received?
    - None
    - 1
    - 2-5
    - 6-10
    - >10
21. Please check if the patient received any of the following supplements.
    - SAMe
    - Lysine
    - Arginine/ornithine
    - Proline
    - Glutathione
    - Zinc
    - Omega-3 fatty acid
    - Patient did not receive any supplements
    - Other
22. Did the patient receive any additional supplements? If yes please list them:
23. Please provide any other comments or information.
