# Supplementary material for: Treatment and outcomes of dogs with hepatocutaneous syndrome or hepatocutaneous‐associated hepatopathy
Source: J Vet Intern Med. 2021 Nov 25;36(1):106–15. doi: 10.1111/jvim.16323 (PMC8783367; doi:10.1111/jvim.16323)
Supplement: Supplementary file 2 — Data S2: Supplementary File 2. [file JVIM-36-106-s001.pdf]

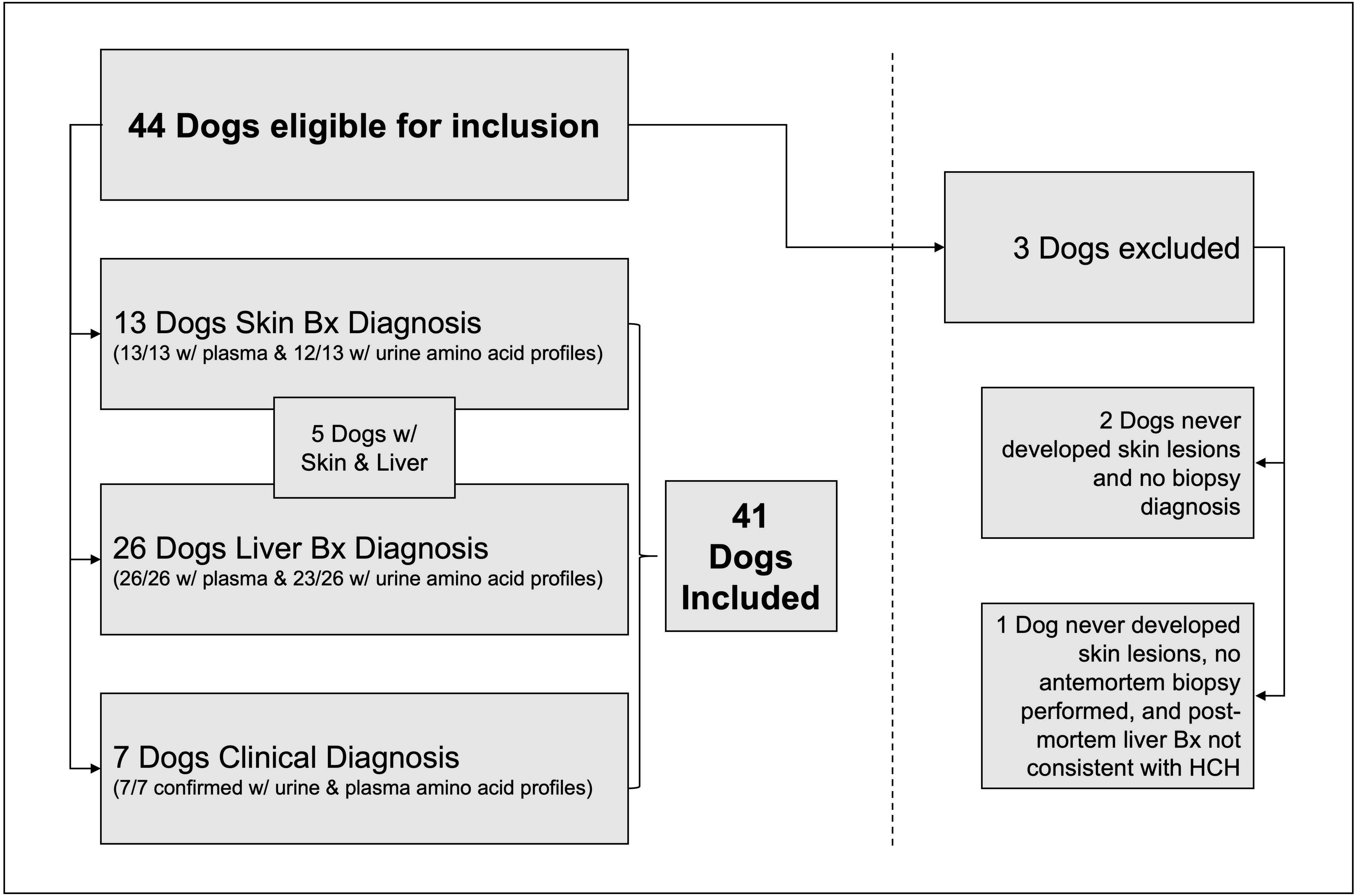
44 Dogs eligible for inclusion
13 Dogs Skin Bx Diagnosis
(13/13 w/ plasma & 12/13 w/ urine amino acid profiles)
5 Dogs w/ Skin & Liver
26 Dogs Liver Bx Diagnosis
(26/26 w/ plasma & 23/26 w/ urine amino acid profiles)
7 Dogs Clinical Diagnosis
(7/7 confirmed w/ urine & plasma amino acid profiles)
41 Dogs Included
3 Dogs excluded
2 Dogs never developed skin lesions and no biopsy diagnosis
1 Dog never developed skin lesions, no antemortem biopsy performed, and post-mortem liver Bx not consistent with HCH
