# Supplementary material for: Treatment and outcomes of dogs with hepatocutaneous syndrome or hepatocutaneous‐associated hepatopathy
Source: J Vet Intern Med. 2021 Nov 25;36(1):106–15. doi: 10.1111/jvim.16323 (PMC8783367; doi:10.1111/jvim.16323)
Supplement: Supplementary file 3 — Table S1: Patient demographics, clinical features, and survival information. [file JVIM-36-106-s002.pdf]

Supplementary Table 1. Patient demographics, clinical features and survival information.

Patient Information										
Patient	Age (yr)	Sex	Breed	Weight (kg)	Diabetic	Skin Lesions at Diagnosis	Skin Lesions Anytime	Survival Interval (days)	Treatment Group**	Nature of Survival Event
1	11	MN	Australian Shepherd	22	NO	FULMINANT	FULMINANT	12	V	Euthanized: ACHES/SND
2	11	FS	Bichon Frise	9.1	NO	NONE	FULMINANT	214	V	Euthanized: ACHES/SND
3	10	FS	Chihuaha	5.5	YES*	NONE	FULMINANT	834	V	Alive/Censored
4	14	MN	Chihuaha	2.3	NO	FULMINANT	FULMINANT	693	V	Euthanized: other (septic abdomen)
5	10	MN	Chihuaha	6.9	NO	NONE	NONE	108	V	Euthanized: other (AKI, presumptive thrombosis)
6	10	FS	CKCS	8	NO	FULMINANT	FULMINANT	131	V	Euthanized: ACHES/SND
7	9	M	Cocker Spaniel	13	NO	MILD	MILD	1126	V	Alive/Censored
8	9	MN	Cocker Spaniel	14	NO	FULMINANT	FULMINANT	359	V	Euthanized: ACHES/SND
9	11	FS	Cocker Spaniel	10.7	NO	MILD	FULMINANT	667	V	Alive/Censored
10	12	MN	Cocker Spaniel	11.5	YES*	NONE	FULMINANT	470	V	Euthanized: ACHES/SND
11	NR	FS	GSD	40.5	YES	FULMINANT	FULMINANT	203	V	Euthanized: ACHES/SND
12	11	MN	GSD Mix	23	NO	FULMINANT	FULMINANT	107	V	Euthanized: ACHES/SND
13	10	MN	GSP	38	YES	MILD	MILD	846	V	Euthanized: other (pneumonia)
14	10	FS	Husky/Lab Mix	33.5	NO	FULMINANT	FULMINANT	498	O	Euthanized: other (multifactorial geriatric problems)
15	12	MN	Labrador Mixed	27	NO	NONE	NONE	157	O	Euthanized: ACHES/SND
16	10	FS	Labrador Retr.	25.8	NO	NONE	NONE	531	V	Alive/Censored
17	7	FS	Labrador Retr.	35	NO	MILD	FULMINANT	523	V	Alive/Censored
18	9	MN	Maltese	4	YES*	NONE	NONE	1488	V	Euthanized: other (hypoglycemia, neurologic signs)
19	8	FS	Maltese	6.6	YES*	FULMINANT	FULMINANT	1783	O	Alive/Censored
20	11	MN	Maltese	9.8	YES*	NONE	FULMINANT	327	V	Euthanized: ACHES/SND
21	9	MN	Mixed Breed	5	NO	NONE	NONE	1443	O	Alive/Censored
22	11	MN	Mixed Breed	27.6	NO	NONE	NONE	346	O	Euthanized: other (multifactorial geriatric problems)
23	11	MN	Poodle Mix	11	NO	FULMINANT	FULMINANT	1065	O	Euthanized: other (abdominal mass, anemia)
24	5	FS	Schipperke	9.3	NO	FULMINANT	FULMINANT	103	V	Euthanized: ACHES/SND
25	8	MN	Shetland Sheepdog	13.6	NO	MILD	FULMINANT	557	V	Euthanized: ACHES/SND
26	14	MN	Shetland Sheepdog	12.1	NO	FULMINANT	FULMINANT	176	O	Euthanized: other (AKI)
27	5	MN	Shetland Sheepdog	17.9	YES*	FULMINANT	FULMINANT	735	O	Euthanized: other (multifactorial disease)
28	12	MN	Shetland Sheepdog	12.7	NO	NONE	FULMINANT	176	V	Euthanized: ACHES/SND
29	10	FS	Shih Tzu	6	NO	FULMINANT	FULMINANT	732	V	Euthanized: ACHES/SND
30	8	FS	Shih Tzu	4.8	NO	FULMINANT	FULMINANT	168	V	Euthanized: ACHES/SND
31	5	MN	Shih Tzu	5	NO	FULMINANT	FULMINANT	1	V	Euthanized: ACHES/SND
32	12	MN	Shih Tzu	7.8	YES	MILD	MILD	17	V	Euthanized: ACHES/SND
33	13	FS	Shih Tzu	6.6	NO	FULMINANT	FULMINANT	56	V	Euthanized: ACHES/SND
34	8	MN	St. Bull Terrier	26.2	NO	FULMINANT	FULMINANT	143	V	Euthanized: ACHES/SND
35	13	FS	Terrier Mix	11.3	NO	FULMINANT	FULMINANT	68	V	Alive/Censored
36	NR	MN	WHWT	8	NO	NONE	NONE	24	V	Alive/Censored
37	11	MN	WHWT	10.3	NO	FULMINANT	FULMINANT	662	V	Euthanized: other (pneumonia, liver mass)
38	9	MN	WHWT	7.5	NO	FULMINANT	FULMINANT	6	V	Died: ACHES/SND
39	8	MN	WHWT	9.6	YES	MILD	MILD	1714	V	Alive/Censored
40	9	MN	WHWT	10.4	NO	FULMINANT	FULMINANT	8	V	Euthanized: ACHES/SND
41	11	MN	WHWT	9.2	YES	FULMINANT	FULMINANT	238	O	Alive/Censored

*Developed diabetes after ACHES/SND diagnosis

**Treatment groups: O = Optimally, V = Variably

FS = female spayed, MN = male neutered, CKCS = Cavelier King Charles Spaniel, GSD = German Shepherd Dog, GSP = German Shorthaired Pointer, Retr. = retriever, St. = Staffordshire, WHWT = West Highland White Terrier, ACHES = Aminoaciduric Canine Hypoaminoacidemic Hepatopathy Syndrome, AKI = Acute Kidney Injury, SND = Superficial Necrolytic Dermatitis
